# Mechanisms Influencing the Spread of a Native Marine Alga

**DOI:** 10.1371/journal.pone.0094647

**Published:** 2014-04-10

**Authors:** Dilys Zhang, Tim M. Glasby, Peter J. Ralph, Paul E. Gribben

**Affiliations:** 1 Plant Functional Biology and Climate Change Cluster, University of Technology, Sydney, New South Wales, Australia; 2 New South Wales Department of Primary Industries, Port Stephens Fisheries Institute, Nelson Bay, New South Wales, Australia; Manchester University, United Kingdom

## Abstract

Like invasive macrophytes, some native macrophytes are spreading rapidly with consequences for community structure. There is evidence that the native alga *Caulerpa filiformis* is spreading along intertidal rocky shores in New South Wales, Australia, seemingly at the expense of native *Sargassum* spp. We experimentally investigated the role physical disturbance plays in the spread of *C. filiformis* and its possible consequences for *Sargassum* spp. Cleared patches within beds of *C. filiformis* (*Caulerpa* habitat) or *Sargassum* spp. (*Sargassum* habitat) at multiple sites showed that *C. filiformis* had significantly higher recruitment (via propagules) into its own habitat. The recruitment of *Sargassum* spp. to *Caulerpa* habitat was rare, possibly due in part to sediment accretion within *Caulerpa* habitat. Diversity of newly recruited epibiotic assemblages within *Caulerpa* habitat was significantly less than in *Sargassum* habitat. In addition, more *C. filiformis* than *Sargassum* spp. recruited to *Sargassum* habitat at some sites. On common boundaries between these two macroalgae, the vegetative growth of adjacent *C. filiformis* into cleared patches was significantly higher than for adjacent *Sargassum* spp. In both experiments, results were largely independent of the size of disturbance (clearing). Lastly, we used PAM fluorometry to show that the photosynthetic condition of *Sargassum* spp. fronds adjacent to *C. filiformis* was generally suppressed relative to those distant from *C. filiformis.* Thus, physical disturbance, combined with invasive traits (e.g. high levels of recruitment and vegetative growth) most likely facilitate the spread of *C. filiformis,* with the ramifications being lower epibiotic diversity and possibly reduced photosynthetic condition of co-occurring native macrophytes.

## Introduction

The spread of introduced invasive plants can have severe impacts on biodiversity [1]–[3]. Similarly, some native plants are also undergoing range expansions and/or becoming more abundant [4]–[6]. There is evidence that range expansions and increasing abundances of native species can have ecological effects as great as those of introduced species [7]–[9]. Although they have received much less attention than their exotic counterparts, the spread of native macrophytes can result in homogenisation of vegetation, and altered community structure and diversity [10]–[12]. The spread of native species into new areas (range shifts) can be considered the ecological analogue of an invasion by introduced species because both result in a species being introduced into a new environment [8]. However, in some instances where native species are spreading and becoming more abundant in areas where they naturally occur, those natives were previously subdominant members of the community. We may expect this to happen when changes to environmental conditions (biotic or abiotic) positively affect the previously sub-dominant species or, negatively affect the previously dominant species, or some combination of the two.

In space-limited environments, the creation of space by disturbance can enable the co-existence of functionally similar species (be they natives or introduced species). For example, competitively inferior species can establish and spread by exploiting newly created space [13]–[15]. But equally, competitively superior species can themselves be prevented from becoming or remaining dominant due to disturbances increasing their mortality or limiting their productivity [16]–[19]. Changes in disturbance regimes appear to be a key mechanism underpinning the spread and increase in abundance of some native plants. For example, changes in temperature (mean or range), nutrient inputs, frequency of fires or herbivore (grazer) abundance can result in the rapid expansion and increases in abundance of previously subordinate community members see [4] for review.

On marine rocky-shores, space is a primary limiting resource, and disturbances that create space can promote the spread of invasive species. For example, the physical removal of native kelp allows colonisation of substrata by the invasive alga *Undaria pinnatifida* [20]. Colonisation of kelp beds was also dependent on the size of the disturbed patch. In the Mediterranean, anthropogenic disturbances (e.g. nutrients and sediment) negatively affect native kelp, promoting the development of turfing macrophytes which facilitate the colonisation of the invasive green alga *C. racemosa* [21]–[23]. *C. racemosa* does not appear to colonise intact kelp beds [24]. Thus, while the spread of native marine macrophytes has been linked to changes in climatic conditions e.g. [5], we may also expect them to spread in areas prone to abiotic disturbance, or where competitors have been removed e.g. [25]. Generally, however, little is known about the conditions that promote the spread of native marine macrophytes.

**Figure 1:**
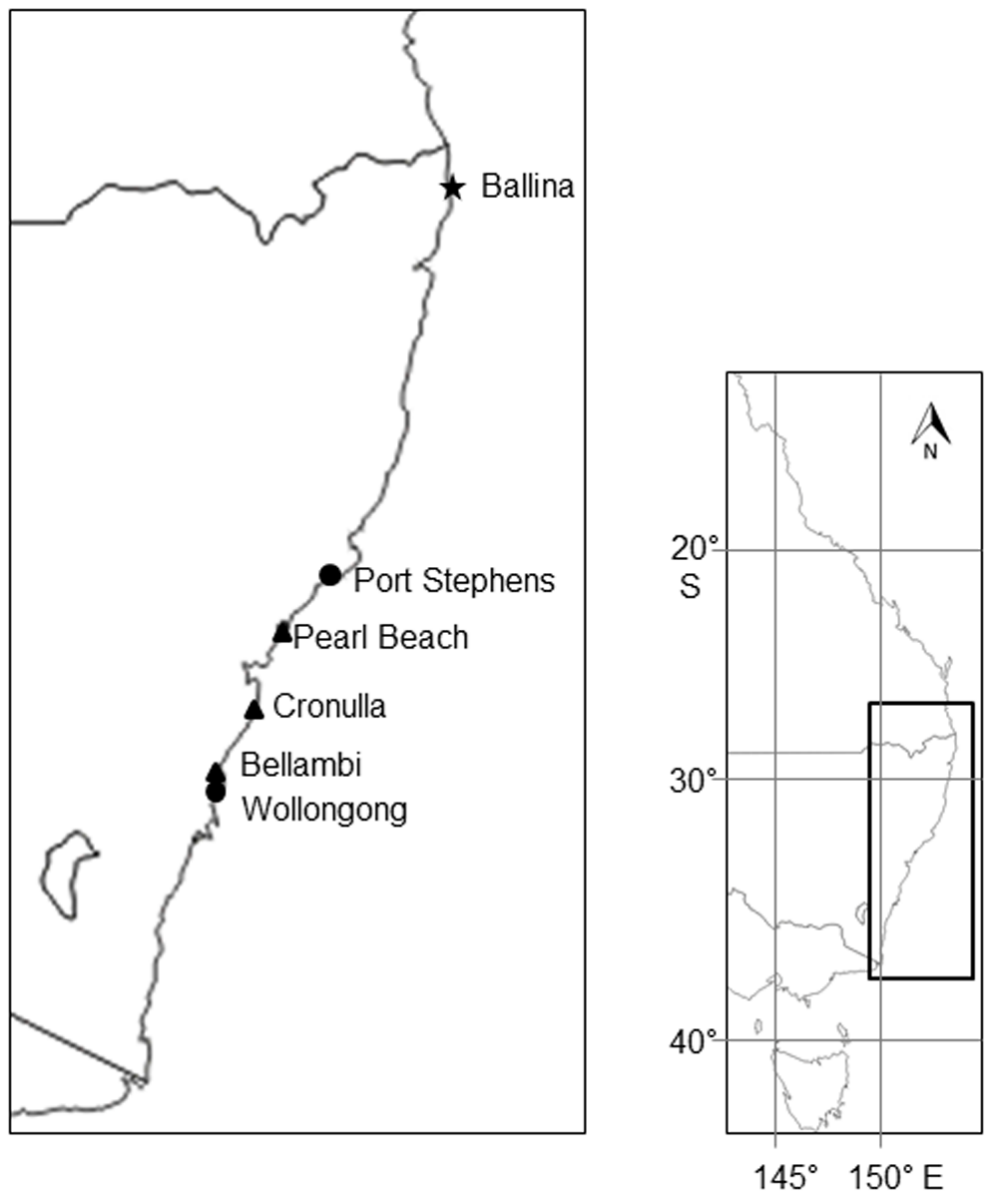
Study area along the east coast of Australia, showing 1) sites where sampling and experiments were conducted (Pearl Beach, Cronulla and Bellambi; black triangles), 2) the historical distribution of *Caulerpa filiformis* (Port Stephens to Wollongong; black circles) and 3) Ballina (black star), the site furthest north where *C. filiformis* has been documented.

In New South Wales, Australia, the loss of habitat-forming macrophytes [26] appears to coincide with the spread of the native green alga, *Caulerpa filiformis* (Family Caulerpaceae). *C. filiformis* was first recorded from Botany Bay and Port Jackson [27] although its distribution is considered to be restricted to a 260 km range from Port Stephens to Wollongong [28] (Fig. 1). The supposed proliferation of *C. filiformis* within its range was first noted during the 1970s [29]. More recently, populations have been recorded at locations as far as 350 km north of its previously recorded northern limit (i.e. at Ballina; Glasby unpublished data) (Fig. 1). The decline of some macrophytes has been attributed to the disturbance effects of urbanisation, such as polluted runoff and historical sewage outfalls [26], [30], [31], yet species of *Caulerpa* can proliferate under such conditions [23], [32]. Thus, *C. filiformis* may be replacing species that are being lost (possibly due to a variety of mechanisms) by occupying newly created space. The spread of *C. filiformis* may have severe implications for the structure and diversity of near-shore coastal communities because *C. filiformis* can form large mono-specific stands (Zhang pers. obs.), is chemically defended and unpalatable to several herbivores, and is structurally simpler than common co-occurring macroalgal species [33]. Structural complexity of a habitat is often positively associated with the diversity of invertebrates e.g. [34], [35]. In addition, once established, *C. filiformis* may also affect the health of competitors it interacts with (e.g. via alleopathy, competition for resources, altering abiotic processes; see [3] for review of invasive plant impacts] further aiding its own spread and increasing its impacts.

In this study we investigated the potential mechanisms underpinning the spread of *C. filiformis.* We focused on its interactions with one of the dominant brown algal complexes in the mid to low intertidal, namely *Sargassum* spp. We tested two hypotheses relating to colonization of space created by a disturbance. First that *C. filiformis* would colonize space, via the recruitment of progagules, faster than *Sargassum* spp., regardless of whether that space was created within beds of *Sargassum* spp. (hereafter, *Sargassum* habitat) or within beds of *C. filiformis* (hereafter, *Caulerpa* habitat). Second, in patches created on boundaries between *Sargassum* habitat and *Caulerpa* habitat, *C. filiformis* would colonise the space via vegetative growth faster than *Sargassum* spp. Both of these mechanisms of colonisation can contribute to high demographic rates and the spread of opportunistic species [36]. We also hypothesized that epibiotic assemblages in newly colonized patches in *Caulerpa* habitat would be less diverse than those within *Sargassum* habitat. These three hypotheses were tested by mimicking physical disturbance at multiple sites. We created patches of different sizes because patterns of colonization, and hence assemblage structure, can vary significantly with patch size [37]. We predicted that patch size would influence the magnitude of differences between habitats. Finally, we hypothesized that *C. filiformis* would negatively affect the health of *Sargassum* spp. and tested this by comparing the photosynthetic ability of *Sargassum* spp. where it interacted with *C. filiformis* versus areas where it did not. We tested this latter hypothesis as invasive macrophytes can have sublethal effects on native species that cannot be detected by population level studies e.g. [1], [2], [38], [39].

## Materials and Methods

### Study species and sites

All sites and organisms sampled were conducted under Permit No. P09/0058-1.0 issued by New South Wales Department of Primary Industries. The field studies did not involve endangered or protected species.

*Caulerpa filiformis* is a green alga found on exposed intertidal and subtidal reefs between 0–6 m water depth in NSW [40] where it grows on a variety of substrata, from rocks to sand [41]. Its root-like rhizomes form dense, entangling mats and give rise to flattened blades with cylindrical, annulated bases. The blades can grow to >40 cm [42]. In NSW, this species is becoming more conspicuous within its range between Port Stephens and Wollongong and is now a dominant intertidal/subtidal habitat-forming species on many rocky shores around Sydney [40], [43], [44]. Observations suggest that it is now also expanding its geographic range (Zhang and Glasby pers obs).

*Sargassum* is a ubiquitous genus of brown algae in Australian waters [45]. Numerous species have been described and accurate identification is difficult, being based on the size and shape of receptacles [28]. We note that the *Sargassum* spp. at each site appeared morphologically similar and the invasive *S. muticum* has not been recorded in Australia. We use only the generic name here but suggest that the most likely species are *S. linearifolium*, *S. verruculosum*, *S. fallax* or possibly *S. vestitum*.

Clearance experiments were replicated at three sites (Pearl Beach 33°32′56.29″S 151°18′29.54″E, Cronulla 34°04′10.01″S 151°09′20.34″E and Bellambi 34°22′07.70″S 150°55′49.57″E) spanning a broad range of *C. filiformis*' distribution (Fig. 1). *C. filiformis* was most abundant (and widely dispersed) at Bellambi and least abundant at Pearl Beach (where it was restricted to just one section of the rock platform), while *Sargassum* spp. were common at each site. The experiment on the effects of *C. filiformis* on the health of *Sargassum* spp. was replicated at just two sites (Cronulla and Bellambi). Although the timing of reproduction for *Sargassum* spp. at our study sites is unknown, *Sargassum* spp. on the east and south coasts of Australia are reproductive from spring to late summer coinciding with the timing of this study (see below) [46], [47].

### Does physical disturbance facilitate the recruitment of propagules of *Caulerpa filiformis* compared to *Sargassum* spp.?

To determine whether physical disturbance facilitates the establishment of *C. filiformis* via recruitment of propagules (i.e. fragments), we created clearings of different sizes (small, 10×10 cm; medium, 20×20 cm; large 40×40 cm) in habitats consisting of *Caulerpa* habitat or *Sargassum* habitat in wave-exposed areas on intertidal rocky shores. Recruitment is defined as a recruit that has settled, survived and grown to become visible to an investigator when sampled [48]. Clearings were made in beds of the different macrophytes that were at least 2 m^2^ and at least 1 m apart (rock pools were avoided). The different types of clearings were interspersed to avoid spatially confounding effects, with n = 4 replicates/treatment/habitat.

Clearance treatments were created using paint scrapers to remove all macro-organisms during 22^nd^ to 29^th^ November, 2011. Plots were inspected and groomed fortnightly to prevent vegetative growth of macrophytes into plots. After 4 months, the percent covers of bare substratum, sand and all sessile biota (primarily algae, sponges and polychaetes) that had recruited into each patch were determined using a grid of 100 regularly spaced points. Total percent cover was determined, not just cover occupied by the point where a recruit attached to the rock platform. Different sized quadrats with different size grids were used to standardize sampling effort within each disturbance treatment (small, 1×1 cm grids; medium, 2×2 cm grids; large, 4×4 cm grids). Sites were sampled in the order that they were established so clearings were in place for the same length of time.

### Is the vegetative growth of *Caulerpa filiformis* into cleared patches higher than for *Sargassum* spp.?

Here we tested whether high rates of vegetative growth enabled *C. filiformis* to colonise bare space more rapidly than *Sargassum* spp. We created the same clearance treatments as described above, but cleared patches were placed on the common boundary between adjacent *C. filiformis* and *Sargassum* spp. patches. The experiment was replicated at the same three sites described above (n = 4 replicates/disturbance treatments/site). The minimum size of patches in contact was the same as described above. Cleared patches were set up at the same time as the experiment, however, for this experiment, vegetative growth of surrounding *Sargassum* spp. and *C. filiformis* was not removed from clearings. After 4 mo, percent cover of both species was determined as described above. Any recruitment via propagules of either species into the clearings that was not obviously due to encroachment (i.e. the appearance of new individuals in the plot clearly not attached to encroaching algae) was not counted.

### Is *Caulerpa filiformis* adversely affecting the health of *Sargassum* spp.?

Chlorophyll fluorescence measurements change with exposure to stress [49] and can be used to infer changes to the photosynthetic efficiency of a plant [50]. Therefore, we used chlorophyll fluorescence to investigate whether *C. filiformis* was having negative effects on the health of *Sargassum* spp. At two sites (Cronulla and Bellambi), we compared chlorophyll fluorescence of *Sargassum* spp. fronds at the edges of beds that were either in direct contact with *C. filiformis* or distant from *C. filiformis* fronds. *Sargassum* spp. collected from edges not in contact with *C. filiformis* were at least 1 m away from *C. filiformis* patches and were often against coralline algae, bare substrata, or the brown alga *Hormosira banksii.* Sites were sampled on different days in March, 2012, with all replicates from a single site collected and measured (details below) in one day. At each site, a single frond was haphazardly selected at the edge of *Sargassum* spp. patches adjacent and distant from *C. filiformis* (n = 15 patches/treatment). Five measures were taken for each frond to account for within frond variation, with the average value being used in analyses.

### Measurements of chlorophyll fluorescence

Initially, we measured chlorophyll fluorescence in the field, however the measurements were highly variable (data not shown) - chlorophyll fluorescence can vary greatly temporally and spatially due to the effects of background abiotic conditions and diel responses of the plants [51]. In addition, macrophytes have to be dark-adapted for at least 30 minutes for accurate readings of F_0_ and F^0^_m_ which was difficult to do in the field. Therefore we developed the following standard procedures. Once collected, *Sargassum* spp. fronds from a single patch (fronds collected adjacent and distant from *C. filiformis* were kept separate) were placed in a perforated bag and submerged in seawater inside a dark, aerated cooler during transit to the laboratory (max. 2 hrs). All collections took place between 07:00 and 12:00 to minimise diel variation in chlorophyll fluorescence [52]. At the laboratory, fronds were kept separate in clear aerated holding tanks (12×20×15 cm) filled with ∼3 cm of filtered (0.2 μm) seawater. Water temperatures in the tanks were maintained between 24–26°C (comparable to field temperatures at time of collection). Using a Diving-PAM (Walz GmbH Effeltrich, Germany: settings; ML int 8, G 12, SP int 12, SP width 0.8s) with a 2 mm plastic fibre, the maximum quantum yield of PSII (Fv/Fm  =  [Fm-Fo]/Fm; where Fm is dark-adapted maximum and Fo is minimum fluorescence) was measured on the dark-adapted samples. The fronds were then light-adapted for 30 min under 400–500 μmol photon m^−2^ s^−1^ irradiance; supplied by 400 W metal halide lamps fitted with a diffuser. Effective quantum yield of PSII (Φ_PSII_  =  [Fm'-Ft]/Fm'; where Fm' is light-adapted maximum and Ft is minimum fluorescence) was estimated using the Diving-PAM. Non-photochemical quenching was determined according to the following equation; NPQ  =  [Fm-Fm']/Fm'. NPQ is a response to protect the plants photosystems from excess light energy or environmental stress [53].

### Statistical analyses

To create direct tests for differences between the recruitment (via propagules) of *C. filiformis* and *Sargassum* spp., we randomly selected two of the four replicate patches to use for each variable (thereby ensuring the data were independent). These data were analysed with orthogonal four factor Analyses of Variance (ANOVA) to determine the effects of patch size (fixed factor with 3 levels; small, medium or large clearings), habitat (fixed factor with 2 levels; *Caulerpa* or *Sargassum* habitat), site (random factor with 3 levels) and a factor termed SvC (i.e., species recruiting, *Sargassum* vs *Caulerpa*, fixed) (n = 2). For all analyses, assumptions of ANOVAs were checked by examining distributions of residuals and plots of residuals vs. means (Quinn & Keough 2002). Non-significant interaction terms were pooled with the residual. SNK post-hoc tests were used to test for differences among levels of significant factors.

Because non-significant results for the factor patch in the analysis above (result not presented) may have been due to small samples sizes (n = 2), we conducted separate three factor orthogonal ANOVAs to provide more robust tests of the effects of patch size (fixed factor with 3 levels; small, medium or large clearings), habitat (fixed factor with 2 levels; *Caulerpa* or *Sargassum* habitat) and site (random factor with 3 levels) on the recruitment of *C. filiformis* and *Sargassum* spp. (n = 4). Thus, these analyses did not have a direct comparison of recruitment of *Sargassum* spp. vs recruitment of *C. filiformis*.

Similar to above, a direct test for regrowth of *Sargassum* spp. vs *C. filiformis* was created by randomly selecting two replicates to use for cover estimates of each algal taxon – these data were analysed using a 3 factor orthogonal ANOVA with the factors site (random factor), patch size (fixed factor with 3 levels; small, medium or large clearings) and SvC (i.e., species colonising, *Sargassum* vs *Caulerpa*, fixed). Again, separate orthogonal 2-factor ANOVAs were used to provide more robust tests of the effect of patch size and site on the regrowth of *C. filiformis* and *Sargassum* spp. to cleared patches on the boundary between *Caulerpa* and *Sargassum* habitat.

Epibiotic assemblages (14 variables including algae, sessile invertebrates, plus sand and rock) that colonized disturbed patches of different sizes were compared between habitats and sites using non-parametric permutational multivariate ANOVA PERMANOVA; [54]. The 3-factor design outlined above was used with 9999 permutations of Bray Curtis similarities and Type III sums of squares. Non-significant interaction terms (P>0.25) were pooled with the Residual to increase the power of tests for other terms in the model. SIMPER was used to identify the variables responsible for differences among factors. Diversity measures (total number of taxa and Shannon diversity index) were compared using the same PERMANOVA design, but based on Euclidean distances among samples.

The effect of *C. filiformis* on the chlorophyll fluorescence of *Sargassum* spp. was analysed using orthogonal 2-factor ANOVA with factors site (random factor with two levels), and position (fixed factor with two levels; adjacent to or distant from *C. filiformis*) with n = 15 replicates/treatment/site. Separate analyses were done for Φ_PSII_ and NPQ. Non-significant interaction terms (*P>*0.25) were pooled with the Residual.

## Results

### Does physical disturbance facilitate the recruitment of propagules of *Caulerpa filiformis* compared to *Sargassum* spp.?

**Figure 2:**
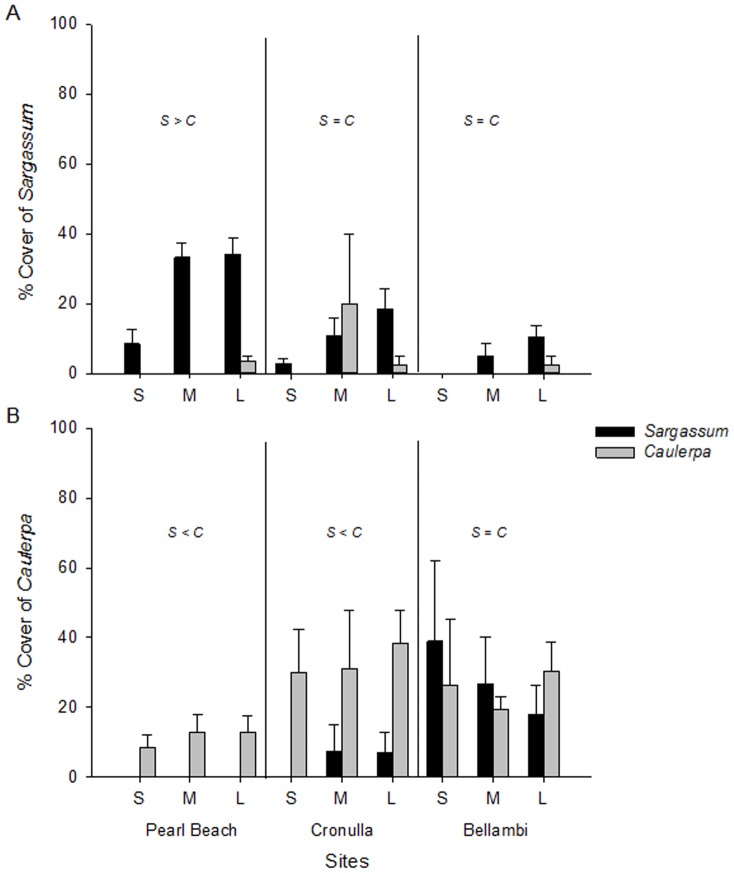
Mean percent cover of recruiting propagules (± SE) of (A) *Sargassum* spp. and (B) *Caulerpa filiformis* into small (S), medium (M) and large (L) cleared patches created in *Sargassum* habitat (black bars) or *Caulerpa* habitat (grey bars) at three sites (Pearl Beach, Cronulla and Bellambi). Letters indicate results of SNK tests comparing differences in recruitment to *Sargassum* spp. habitat (S) or *Caulerpa* habitat (C) per site (n = 4).

Direct comparisons of recruitment of *C. filiformis* vs *Sargassum* spp. (using n = 2 independent replicates) identified significant differences according to habitat (SvC x Habitat *F_1,52_* = 28.18, *P* = 0.0001) and Site (SvC x Site *F_2,52_* = 28.18, *P* = 0.0001). Specifically, recruitment of *C. filiformis* was significantly greater than recruitment of *Sargassum* spp. in *Caulerpa* habitat, and equivalent to *Sargassum* spp. recruitment in *Sargassum* habitat. Recruitment of *C. filiformis* was significantly greater than recruitment of *Sargassum* spp. at two sites, while there was no significant difference at Pearl Beach (although there was a trend for *Sargassum* spp. recruitment to be greater at this site (Fig. 2).

Analysed separately, there were interactive effects of Habitat and Site on recruitment of *Sargassum* spp. (Table 1). Specifically, *Sargassum* spp. recruitment was greater in *Sargassum* habitat (mean ± SE, 25±4.2%) than in *Caulerpa* habitat (1±0.7%) at one site (Pearl Beach), but not significantly different between habitats at the other two sites (where recruitment to each habitat was typically small (6±2%, Fig. 2A)). These differences were reflected in the comparison among sites, with recruitment of *Sargassum* spp. to *Sargassum* habitat being significantly greater at Pearl Beach than at the other two sites, whereas recruitment of *Sargassum* spp. to *Caulerpa* habitat was minimal and did not differ among sites (3±2%, Fig. 2A). Recruitment of *Sargassum* spp. into medium and large patches was significantly greater than recruitment into small patches (Fig. 2A and next section), with the pattern being consistent among sites and between habitats (Table 1).

**Table 1:** ANOVA comparing the effects of disturbance (small, medium or large patches), habitat (*Sargassum* vs *Caulerpa*) and site (random factor) on the recruitment of *Sargassum* spp. (n = 4).

Factor	df	MS	*F*	*P*
Disturbance	2	0.08	6.45	**0.003**
Habitat	1	0.20	2.42	0.260
Site	2	0.06	5.27	0.008
Disturbance x Habitat	2	0.03	1.60	0.309
Disturbance x Site	4	0.01	0.88	0.484
Habitat x Site	2	0.08	6.88	**0.002**
Disturbance x Habitat x Site	4	0.02	1.64	0.179
Residual	54	0.01		

*Disturbance x Site (P>0.25) was pooled with the residual to create the denominator of F tests for other interaction terms in the model. SNK post-hoc tests for the factor Disturbance: Small < Medium  =  Large). Results of Habitat x Site post hoc tests are presented in* *Fig. 2A* *.*

The recruitment of *C. filiformis* also varied interactively by Habitat and Site (Table 2). Specifically, recruitment of *C. filiformis* was significantly greater in *Caulerpa* habitat than in *Sargassum* habitat at two sites, and similar between habitats at the other site (Fig. 2B). At the sites where *C. filiformis* recruitment was greatest in *Caulerpa* habitat, its mean percentage cover was 6±2% and 19±5%, while at the site where *C. filiformis* recruited equally well to both *Sargassum* and *Caulerpa* habitats, its mean cover was 27±5%. The recruitment of *C. filiformis* did not differ significantly among patch size (Table 2).

**Table 2:** ANOVA comparing the effects of disturbance (small, medium or large patches), habitat (*Sargassum* vs *Caulerpa*) and site (random factor) on the recruitment of *Caulerpa filiformis* (n = 4).

Factor	df	MS	*F*	*P*
Disturbance	2	0.001	0.04	0.964
Habitat	1	0.278	1.930	0.299
Site	2	0.268	6.98	0.002
Disturbance x Habitat	2	0.019	0.49	0.615
Disturbance x Site	4	0.017	0.45	0.775
Disturbance x Site	2	0.144	3.74	**0.029**
Disturbance x Habitat x Site	4	0.010	0.27	0.899
Residual	54	0.042		

*Disturbance x Site and Disturbance x Habitat x Site (P>0.25) were pooled with the residual to create the dominator of F tests for all other terms, except Habitat. SNK post-hoc test results for Habitat x Site are presented in* *Fig. 2B* *.*

### Is the vegetative growth of *Caulerpa filiformis* into clearings higher than for *Sargassum* spp?

**Figure 3:**
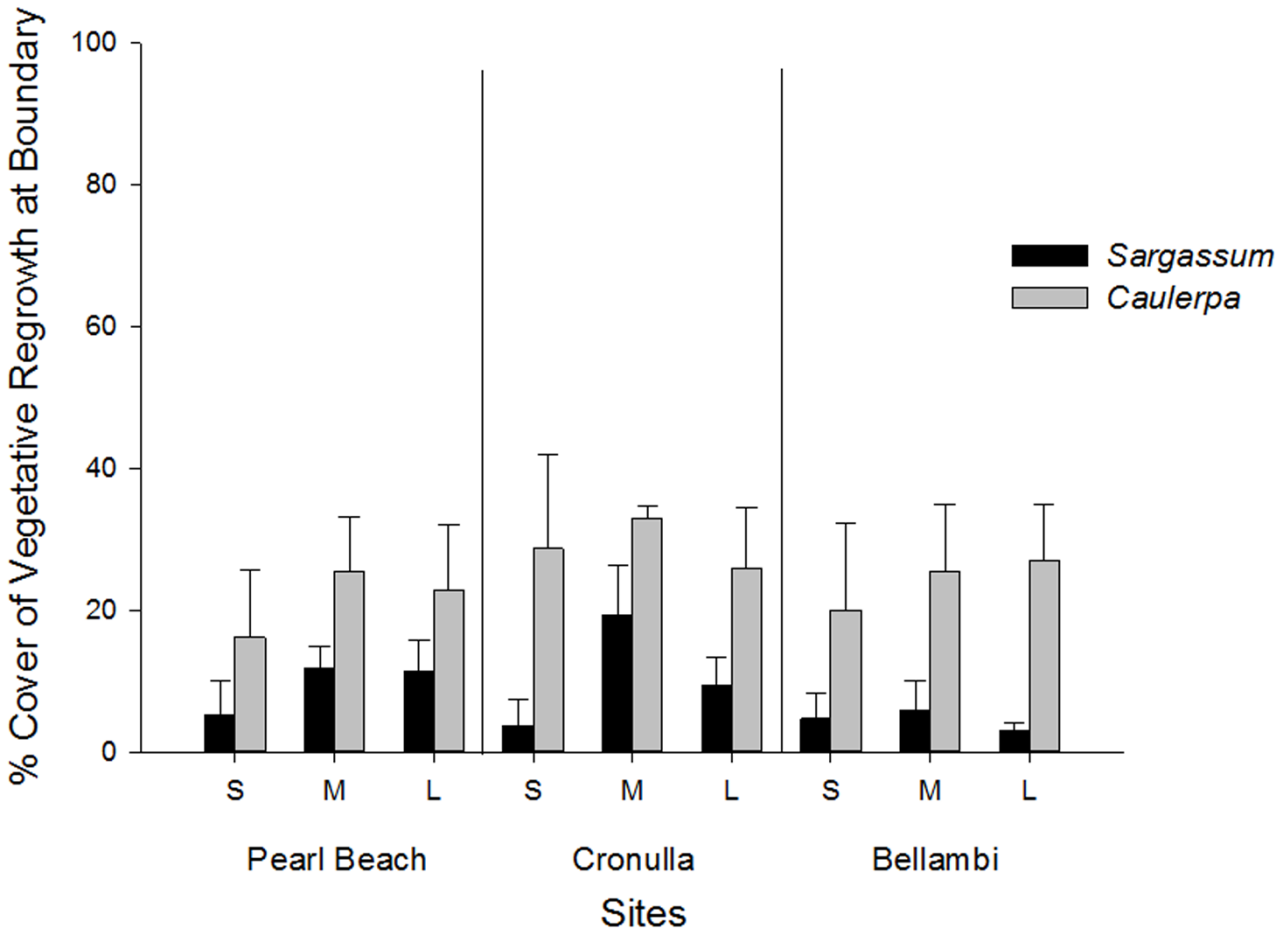
Mean percent cover (± SE) of recolonising (via vegetative growth) *C. filiformis* and *Sargassum* spp. into small (S), medium (M) and large (L) cleared patches created on the boundary of *Sargassum* spp. and *Caulerpa filiformis* habitats at three sites (Pearl Beach, Cronulla and Bellambi). (n = 4 patches/site).

Vegetative growth of *C. filiformis* into patches was significantly greater than recolonisation of *Sargassum* spp. (SvC *F_1,30_* = 32.58, *P* = 0.0001). This result was consistent for all sites and patch sizes. Overall the mean (±SE) percent recolonisation by *C. filiformis* (25±3%) was three times higher than for *Sargassum* spp. (8±1%) (Fig. 3). Using the more robust statistical design (with n = 4 replicates) and analysing the two algal taxa separately, no significant differences in recolonisation of either taxon were detected among patch sizes (*C. filiformis, F_2,31_* = 0.389, *P* = 0.681; *Sargassum* spp., *F_2,31_* = 2.542, *P* = 0.095) or among sites (*C. filiformis, F_2,31_* = 0.598, *P* = 0.556; *Sargassum* spp., *F_2,31_* = 1.826, *P* = 0.178).

### Are assemblages colonising patches in *Caulerpa filiformis* different from those in *Sargassum* spp.?

Epibiotic assemblages that colonised patches after four months differed significantly among patch sizes (Pseudo -*F*_2,64 df_ = 3.49, *P* = 0.0023). SIMPER analyses showed that small patches were characterised by sand, bare rock and *C. filiformis* (together comprising 93% of similarity among replicates). In comparison, medium patches had less bare rock, more brown filamentous algae, coralline algae and *Sargassum* spp. Large patches had the least bare rock, and the most algae (all species). The average cover of *Sargassum* spp. was 2±1% in small patches, 12±4% in medium and 12±3% in large patches, whereas *C. filiformis* cover was consistent among patch sizes (17±6%, 16±4% and 18±4% in small, medium and large patches respectively).

Epibiotic assemblages in patches also varied significantly between habitats at some sites (Habitat x Site Pseudo-*F*_2,64 df_ = 3.09, *P* = 0.0047). Pairwise tests indicated that these newly established assemblages differed between habitats at Pearl Beach (t = 3.79, *P* = 0.0001) and Cronulla (t = 2.14, *P* = 0.019), but not at Bellambi (t = 1.37, *P* = 0.135). SIMPER analyses showed that the taxa driving these differences differed between the two sites, so these were investigated with one way univariate comparisons between habitats. Covers of coralline algae were significantly greater in patches created within *Sargassum* habitat at both Pearl Beach (*F*_1,22 df_ = 4.44, *P* = 0.0047) and Cronulla (*F*_1,22 df_ = 10.03, *P* = 0.0045). Two other algae, *Padina* sp. and *Laurencia* sp. were found only in *Sargassum* habitat at both sites. The only taxon that was consistently more abundant in patches created in *Caulerpa* habitat was *C. filiformis*. At the third site (Bellambi), where newly recruited assemblages did not differ between habitats, all replicate patches were dominated by sand and *C. filiformis* with the two accounting for >85% of similarity among replicates in both habitats.

**Figure 4:**
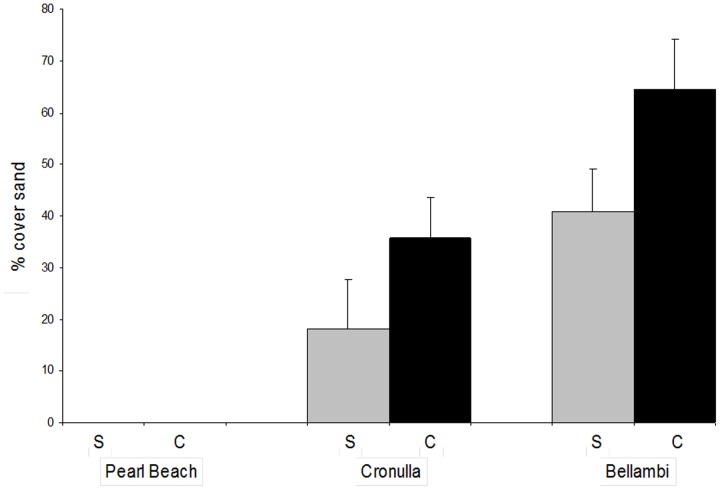
Mean percent cover (± SE) of sand in patches created within *Sargassum* habitat (S, grey bars) or *Caulerpa* habitat (C, black bars) at each of three sites (n = 12, replicates pooled across patch sizes).

The percentage cover of sand in patches differed significantly among sites (*F*_2,64 df_ = 25.16, P = 0.0001; Fig. 4) and was significantly greater in *Caulerpa* habitat than *Sargassum* habitat (*F*_1,64 df_ = 5.22, P = 0.0257).

Total number of taxa and Shannon diversity index showed identical patterns among treatments. Shannon diversity increased significantly (Pseudo-*F*_2,66 df_ = 14.59, P = 0.0001) with patch size (small 0.55< medium 0.76< large 1.04) and was significantly less (Pseudo-F_1,66 df_ = 30.19, P = 0.0001) in patches within *Caulerpa* habitat (0.58) compared to *Sargassum* habitat (0.986). These patterns were consistent among sites.

### Is *Caulerpa filiformis* adversely affecting the health of *Sargassum* spp.?

**Figure 5:**
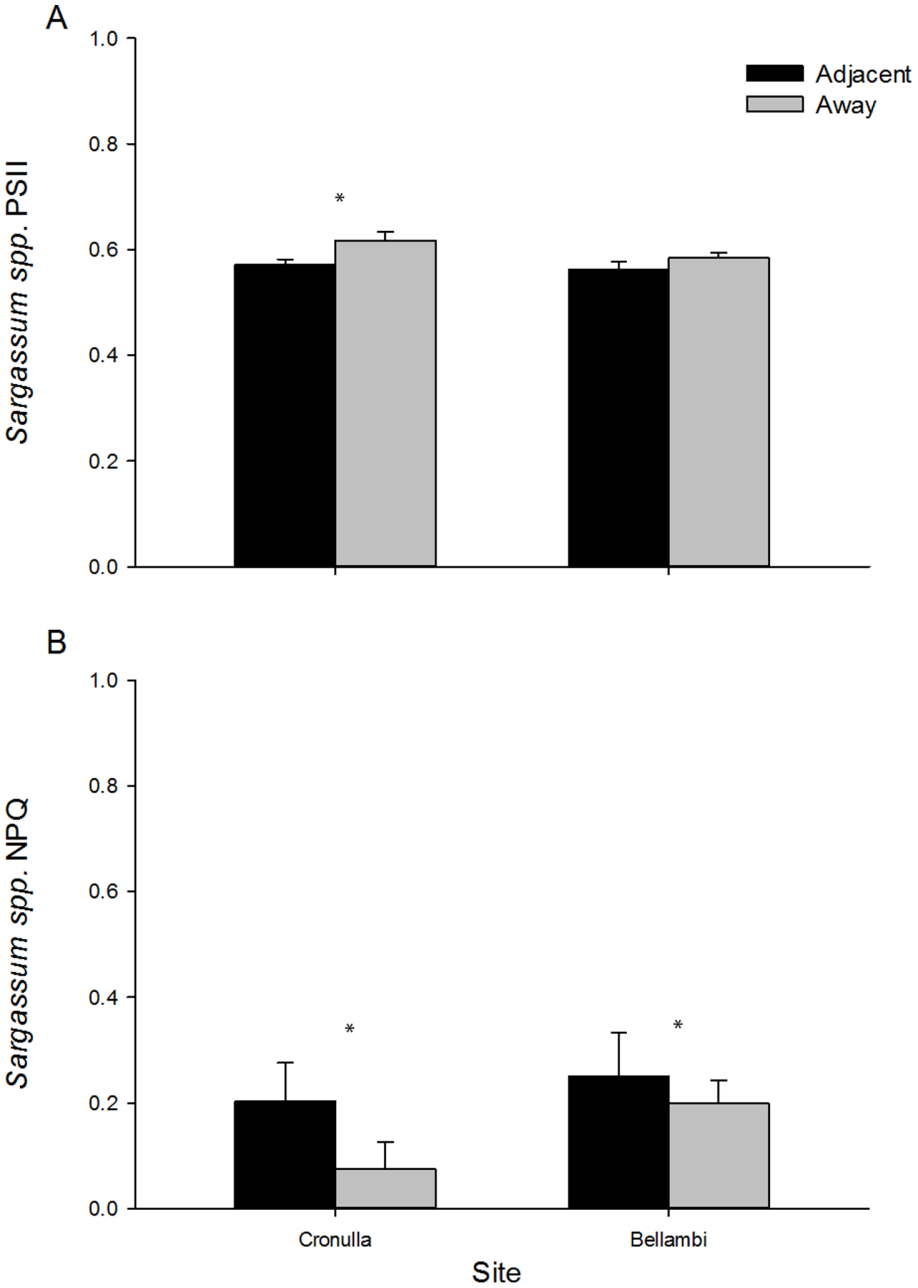
Mean (±SE) phytosynethetic activity of *Sargassum* spp. fronds measured as (A) Φ_PSII_ or (B) NPQ at two sites. Measurements (n = 15 fronds/edge/site) were taken at edges of *Sargassum* habitat which were either adjacent to *Caulerpa* habitat (black bars) or and away from (grey bars). *above bars indicate significant results of SNK comparisons of means.

Photosynthetic activity (Φ_PSII_) differed according to proximity to *Caulerpa* habitat at some sites (Position x Site *F_1,56_* = 42.35, *P* = 0.001). SNK tests showed that Φ_PSII_ of *Sargassum* spp. was significantly lower where it was adjacent to *Caulerpa* habitat at Cronulla, but did not differ between positions at Bellambi (although differences were in the same direction as for Cronulla, Fig. 5A). NPQ measurements of *Sargassum* spp. fronds were significantly higher where they were adjacent to *C. filiformis* compared to edges against other algae and this was consistent at both sites (*F_1,57_* = 11.94, *P* = 0.001; Fig. 5B). Lower Φ_PSII_ and higher NPQ indicates the health of *Sargassum* spp. is reduced in fronds adjacent compared to away from *Caulerpa* habitat.

## Discussion

For native species undergoing range expansions or increases in abundance, we may expect positive responses to disturbance. We found support for our hypothesis that physical disturbance (cleared patches created in established assemblages) promotes the recruitment (at two of three sites) and vegetative spread (at all sites) of *C. filiformis* and, once established, the alga appears to have negative effects on the physiological health of *Sargassum* spp. In addition, newly colonised patches within *Caulerpa* habitat supported a less diverse community compared to patches in *Sargassum* habitat.

Several mechanisms may explain the differences among sites in patterns of recruitment for *C. filiformis* and *Sargassum* spp. In coastal ecosystems, increasing sedimentation can alter macrophyte community structure by removing dominant habitat-forming macrophytes and inhibiting their recruitment success, as well as facilitating sediment tolerant species [55]–[60]. In our study, the percentage cover of sand in cleared patches was lowest at Pearl Beach (sand was absent from patches), the site of highest *Sargassum* spp. recruitment and lowest *C. filiformis* recruitment, intermediate at Cronulla and highest at Bellambi where the recruitment of *C. filiformis* was highest and *Sargassum* spp. very low (Fig. 4). *Caulerpa* spp. may have been more abundant at sandy sites due to greater tolerance to sedimentation than *Sargassum* spp. [61], [62], although some species of *Sargassum* are capable of recruiting to cobbles covered with fine sediment [63]. Macroalgae that are able to trap and bind sediments can benefit from sedimentation [55], [58]. Piazzi et al. [22] showed that, on rocky shores, the invasive alga, *C. racemosa,* was not affected by sedimentation, whereas several native macrophytes were hindered by sediment deposition. Thus, increasing sedimentation may promote the establishment of *C. filiformis.* Indeed, this species has been documented as being positively associated with sand in Australia [29] and South Africa [64]. It is possible that sediment deposition may be an important mechanism of disturbance that is creating space for *C. filiformis*, perhaps in addition to nutrient enrichment as proposed by [29]. In addition, the limited recruitment of *C. filiformis* at Pearl Beach may reflect a lesser propagule supply. Propagule pressure is often a strong driver of the spread of invasive species and also for some marine macrophytes undergoing range expansions, particularly in disturbed environments [25], [65]. Although we did not quantify it in this study, the sizes of intertidal and subtidal populations of *C. filiformis* were smallest at Pearl Beach and largest at Bellambi. The intertidal *Caulerpa* habitat at Pearl Beach was restricted to one large patch while the alga was spread widely across the intertidal rock platform at Bellambi, with a more intermediate distribution at Cronulla. Populations of *C. filiformis* may need to reach a threshold size before propagule supply is large enough to create a positive feedback and accelerated expansion of the population [66].

Habitat surrounding cleared patches (either *Sargassum* or *Caulerpa*) was an important mediator of the recruitment of *Sargassum* spp. and *C. filiformis.* This may relate simply to proximity of adult algae, or may reflect each habitat's ability to trap sediment (possibly promoted by the dense longer fronds of *C. filiformis* compared to *Sargassum* spp. in this study). Secondary metabolites from *C. filiformis* could also have hindered recruitment of *Sargassum* spp. to *Caulerpa* habitat, as grazers may differentially consume *Sargassum* spp. recruits over *C. filiformis* [67], [68]. It is likely that the habitat surrounding cleared patches also affected the diversity of epibiota associated with newly recruited patches, which was always greater within *Sargassum* habitat than within *Caulerpa* habitat. This pattern held even at Bellambi where cleared patches within *Sargassum* habitat were actually colonized by ∼28% *C. filiformis*, which was greater than within *Caulerpa* habitat (20% *C. filiformis* colonization). That is, the significantly greater diversity in the patches within *Sargassum* habitat was most likely due to the surrounding habitat rather than the dominant alga that colonized the patches at this site. *Caulerpa* habitat may be less diverse than *Sargassum* habitat due to the reduced structural complexity of the former, the greater percentage of sand and/or sediment anoxia (and accumulation of toxic sulphides) which negatively affect biota associated with species of *Caulerpa* [39], [69].

Recruitment of algae also varied with level of disturbance (i.e. cleared patch size); small clearances contained lower proportions of *Sargassum* spp. and brown filamentous algae compared to medium and large clearances. Our findings differ from Airoldi [70] who showed that *Sargassum* spp. tended to recruit more to smaller compared to larger cleared patches (although results were not significant). The apparent discrepancy in results most likely relates to the difference patch sizes used in the two studies; Airoldi [70] used patches (150–320 cm^2^) that were intermediate between our small (100 cm^2^) and medium (400 cm^2^) patch sizes. Moreover, recruitment by *Sargassum* spp. in our study (∼5–35%) was far greater in all patch sizes than found by Airoldi (∼1–10%).

Vegetative growth into cleared patches on the boundary between habitats was consistently higher for *C. filiformis* compared to *Sargassum* spp. across all sites and disturbance levels. This suggests that, once established, *C. filiformis* is generally better able to exploit freshly disturbed space via vegetative growth than *Sargassum* spp. This may be because *Sargassum* spp. were more damaged by the process of clearing space and/or because *C. filiformis* can grow faster. The latter explanation is likely as species of *Caulerpa* are known to grow very quickly from all parts stems, leaves and roots; [71]. Fast vegetative growth is a trait typical of opportunistic species [36] and appears to be common for macrophytes in impacted sediments [see 61 for review]. In addition, vegetative growth likely further stabilises sediments facilitating *C. filiformis*' own growth and giving it a competitive advantage over *Sargassum* spp. However, the germinating zygotes of species of *Sargassum* are retained on the fronds before being released with a sticky rhizoid which can facilitate quick (and nearby) attachment e.g. [72].

One aspect we did not address was temporal variation in colonisation of cleared patches. Kennelly [73] found that removal of kelp, leaving only the understorey, resulted in cleared patches colonised by turfs except in winter, when kelp recruitment was high and better able to recolonise space. Although the timing of *Sargassum* spp. reproduction does vary temporally and spatially [74], in our study *C. filfiormis* was able to colonise *Sargassum* habitat at two sites during a period when *Sargassum* spp. were recruiting to cleared patches (i.e. *Sargassum* spp. were reproductively active). This suggests that *Sargassum* spp. may generally be poor competitors for space, which is consistent with Airoldi's findings over 12 months [68]. However, at our Pearl Beach site, where *C. filiformis* did not recruit to *Sargassum* habitat, *Sargassum* recruitment into medium and large patches (34±2.8%) was comparable to the recruitment of *C. filiformis* to *Caulerpa* habitat (29.3±4.7%) and *Sargassum* habitat (27.9±8.9%) at Bellambi. Thus, at some sites (and perhaps times of the year), *Sargassum* spp. may outcompete *C. filiformis* for space, particularly where there is less sediment or a smaller population of *C. filiformis* (e.g. Pearl Beach). Importantly, the loss of habitat-forming macrophytes and associated communities can persist years after disturbance [75]. For example, removal of *Ascophyllum nodosum* canopy (i.e. leaving the understorey in place) resulted in colonisation by two species of *Fucus* for 12 years [76], [77]. Understanding seasonal variation in recruitment dynamics to disturbed patches, and the temporal response of *C. filiformis, Sargassum* spp. and the communities they support post-colonisation, will be an important avenue for future research.

Whilst disturbance is an important mechanism facilitating the initial establishment of opportunistic macrophytes, once established, some can successfully outcompete native species – i.e. they can switch from being passengers to drivers of ecological change [24], [78], [79]. Similarly, in this study we found some evidence for negative effects of the native *C. filiformis* on the physiological health of *Sargassum* spp. This could have resulted from several mechanisms. First, *C. filiformis* could overshade *Sargassum* spp. (*Caulerpa* fronds can reach >40 cm) reducing light levels and limiting its access to essential elements from the water column [80]. However, this seems unlikely because *Sargassum* spp. fronds were typically of a comparable height to *C. filiformis.* Moreover, NPQ usually decreases when macrophytes are shaded as the xanthophyll cycle relaxes [81], yet we measured an increase in NPQ of *Sargassum* spp. fronds that were against *Caulerpa.* Second, *C. filiformis* may alter the abiotic conditions of the trapped sediments by inducing sediment anoxia - dense mats of macrophyte species can cause substrate anoxia via a reduction in photosynthesis, increased algal respiration and detritus accumulation [39], [82], [83] - and/or producing toxic sulphides to which it is tolerant [84]. Third, production of allelochemicals that negatively affect competitors can promote the spread of some macrophytes [85], [86]. Although secondary metabolites (e.g. Caulerpenyne) from *Caulerpa* spp. including *C. filiformis* are unpalatable to most grazers [33], [67], [87], it is not known whether these or other potential allelochemicals could affect *Sargassum* spp. Understanding the mechanisms by which *C. filiformis* is potentially affecting *Sargassum* spp. and the demographic consequences for *Sargassum* spp. warrants further investigation.

Here we have shown that physical disturbance (creation of space) can enhance the recruitment and promote vegetative growth of a native alga, *C. filiformis,* whose potential increase in abundance and spread may have serious consequences for coastal biodiversity. This study was conducted on intertidal rock platforms where *Sargassum* spp. appear to be the main competitors with *C. filiformis*. *C. filiformis* is more common on subtidal reefs (Glasby, unpubl data), where it also co-exists with species of kelp (e.g. *Ecklonia radiata*) and other brown algae (e.g. *Phyllospora comosa*) at several sites throughout its distribution (Gribben, Glasby pers obs). Many of these subtidal brown macrophytes share similar reproductive strategies to *Sargassum* spp. [88]. Thus, following physical disturbance these subtidal habitats may also be susceptible to colonisation by *C. filiformis*. Indeed, *C. filiformis* appears to be replacing these important subtidal habitat-forming macrophytes at several sites throughout its distribution (Gribben, Glasby pers obs). Given growing coastal populations, and predicted increases in physical disturbance in coastal ecosystems (e.g. increased frequency and intensity of storms), these attributes may facilitate an increase in abundance and/or spread of species such as *C. filiformis.* However, further research is required to incorporate broader temporal scales into understanding the consequences of its interactions with native macrophytes and communities more generally.
